# Supplementary material for: A Crucial Role for Ergosterol in Plasma Membrane Composition, Localisation, and Activity of Cdr1p and H+-ATPase in Candida albicans
Source: Microorganisms. 2019 Sep 22;7(10):378. doi: 10.3390/microorganisms7100378 (PMC6843828; doi:10.3390/microorganisms7100378)
Supplement: Supplementary file 1 [file microorganisms-07-00378-s001.pdf]

microorganisms

MDPI

Supplementary Materials

We concluded that auxotrophic phenotype of *erg11Δ/Δ* is suppressed after adding the combination of adenine and uracil, but not aminoacids (Figure S1A). Here, we presented the results using two different sets of aminoacids, neither of them alone restored *erg11Δ/Δ* growth defect on minimal YNB medium. Additionally, due to *ura3Δ/URA3* genotype of KS028 strain we compared the results with *URA3 erg11Δ/Δ* strain (KS058), constructed during this study, according to Materials and Methods (Figure S1B).

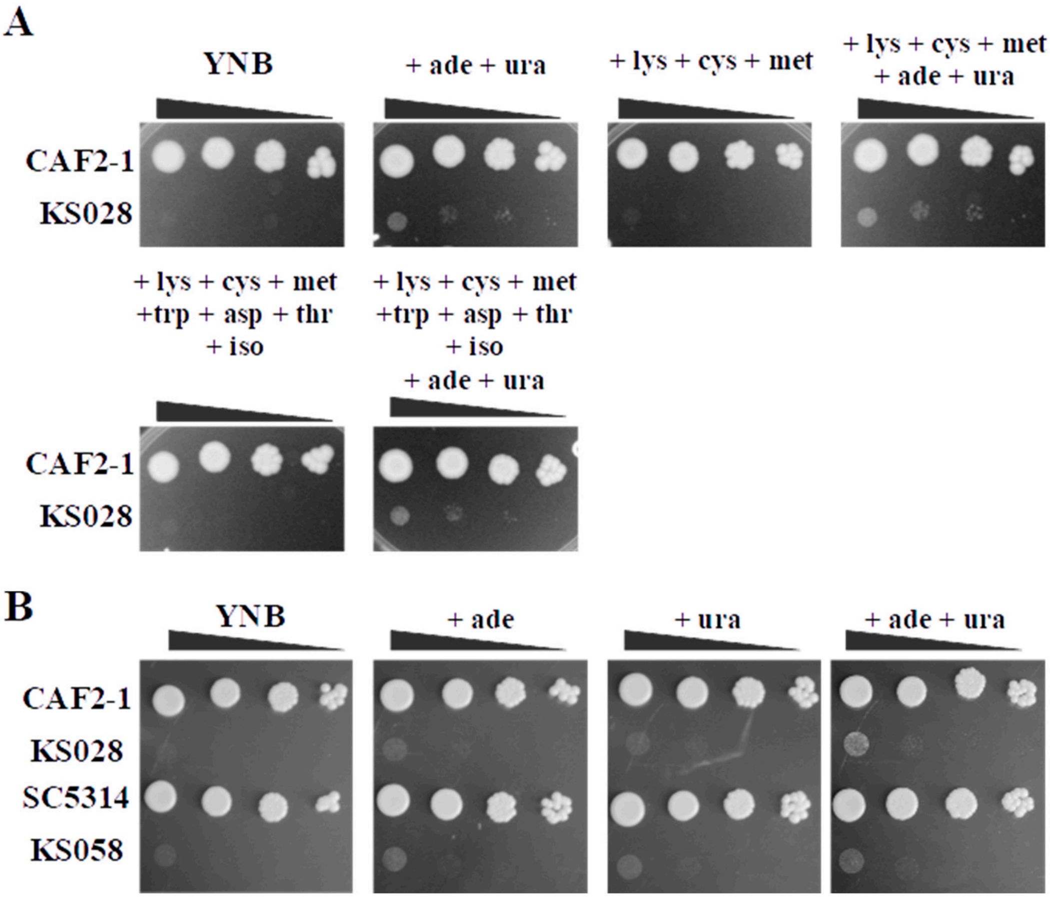

Figure S1. (**A**) Growth phenotypes of *C. albicans* CAF2-1 (WT) and KS028 (*erg11Δ/Δ*) strains after 5 days incubation at 28 °C in minimal YNB medium, supplemented with: adenine (ade), uracil (ura), lysine (lys), cysteine (cys), methionine (met), tryptophan (trp), asparagine (asp), threonine (thr) or isoleucine (iso). (**B**) Growth phenotypes of *C. albicans* CAF2-1 (*ura3Δ/URA3*) and KS028 (*ura3Δ/URA3 erg11Δ/Δ*), SC5314 (*URA3*) and KS058 (*URA3 erg11Δ/Δ*) strains after 3 days incubation at 28 °C in minimal YNB medium, supplemented with: adenine (ade) or uracil (ura).

In our studies we experimented with double knockout strain, *erg3Δ/Δ erg11Δ/Δ* (*C. albicans* DSY1769, a kind gift from Prof. D. Sanglard). We concluded that single *erg11Δ/Δ* knockout (*C. albicans* KS028) display lesser growth defect when cultured on complex YPD medium than DSY1764 strain (Figure S2). For more clear presentation we did not include DSY1751 (*erg3Δ/Δ*) in the graph (Figure S2B).

Microorganisms **2019**, *7*, 378; doi:10.3390/microorganisms7100378 www.mdpi.com/journal/microorganisms

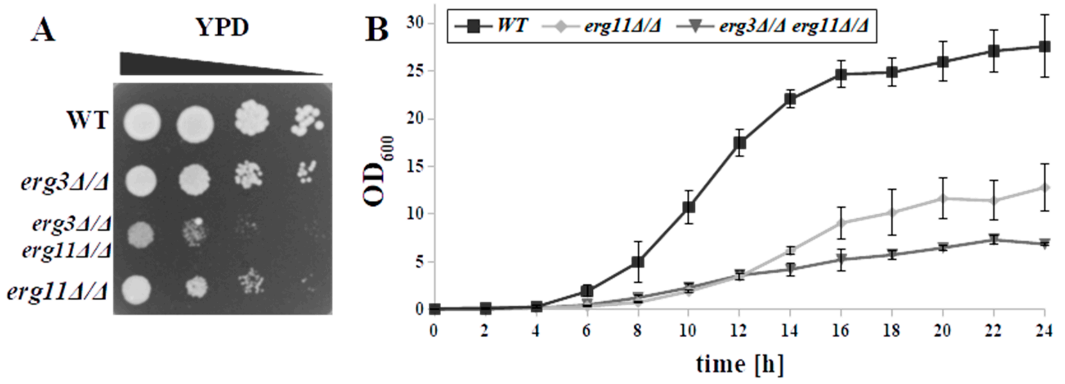

Figure S2. (**A**) Growth phenotypes of *C. albicans* CAF2-1 (WT), KS028 (*erg11Δ/Δ*), DSY1751 (*erg3Δ/Δ*) and DSY1764 (*erg3Δ/Δ erg11Δ/Δ*) strains after 48 h incubation at 28 °C in complex YPD medium. (**B**) Growth curves of *C. albicans* CAF2-1 wild type (WT), KS028 (*erg11Δ/Δ*) and DSY1764 (*erg3Δ/Δ erg11Δ/Δ*) strains in YPD medium (28 °C, 120 rpm, YPD medium).

In our studies we determined small levels of eburicol, 14*α* methylfecosterol and 4*α* methylfecosterol in the strain KS028 (Table S1).

Table S1. Sterol metabolites (μg/mg dry mass of isolated lipids, means ± SD, *n* =3) in *C. albicans* CAF2-1 (WT) and KS028 (*erg11Δ/Δ*) strains.

Strain	Time of culture (hrs)	Eburicol	14*α* methylfecosterol	4*α* methylfecosterol
WT	8	-	-	-
	14	-	-	-
	24	-	-	-
erg11Δ/Δ	8	3.5 ± 0.5	2.3 ± 0.1	3.3 ± 0.1
	14	5.7 ± 3.6	2.3 ± 1.8	1.1 ± 1
	24	3.8 ± 2.3	1.4 ± 0.3	1.8 ± 0.3

Initially we were experimenting with additional time point, early stationary phase (18th hr of growth). The western blot analysis presented in Figures 3 and 4 were cut into separate boxes to present only 8, 14 and 24 hr of growth. In Figure S3 we present both immunoblot analysis with internal control (Ponceau S) included for both western blots from Figures 3 and 4.

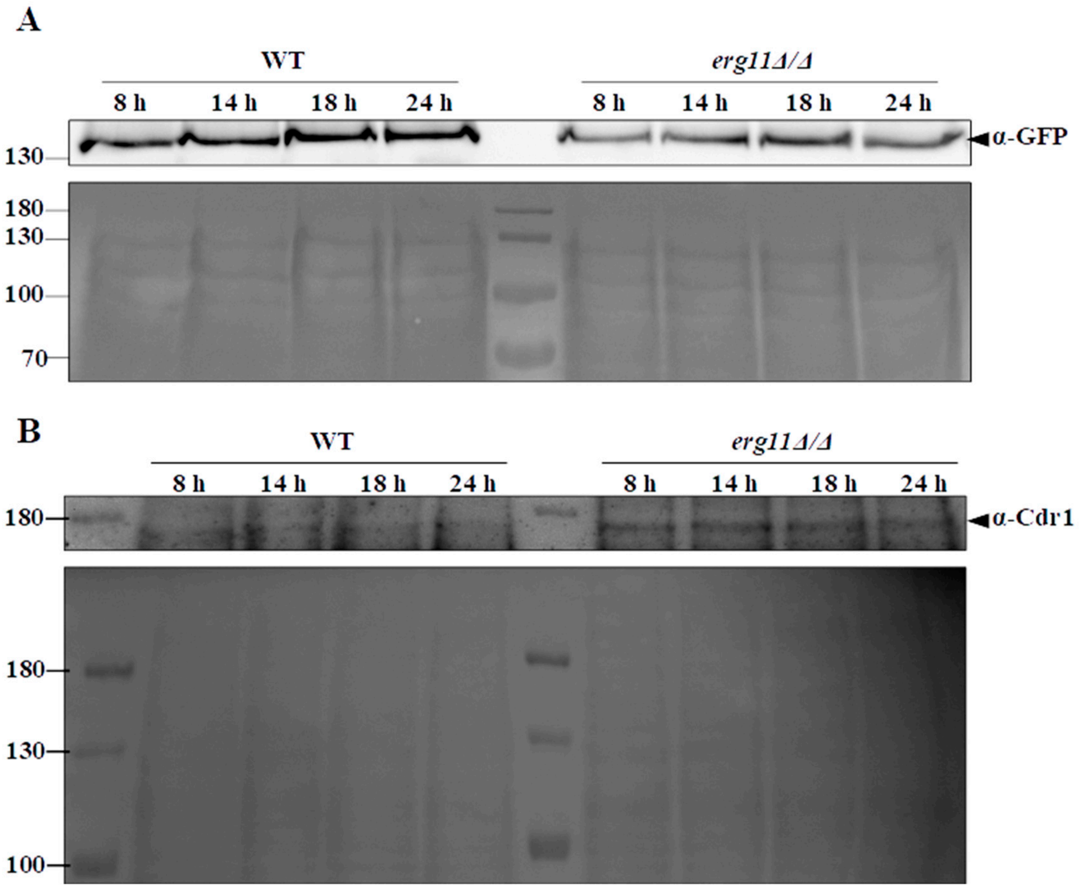

Figure S3. (**A**) Immunoblot analysis of Pma1-GFPp levels in *C. albicans* YHXW11 (WT) and KS045 (*PMA1-GFP*, *erg11Δ/Δ*) strains during growth (8, 14, 18 and 24 h). The samples were resolved on 10% SDS-PAGE gel and probed by an anti-GFP antibody. Ponceau S staining was used as the loading control. Experiment is a representative of three independent assays. (**B**) Immunoblot analysis of Cdr1p levels in *C. albicans* CAF2-1 (WT) and KS028 (*erg11Δ/Δ*) strains during growth (8, 14, 18 and 24 h). The samples were resolved in 6% SDS-PAGE gel and probed by an anti-Cdr1 antibody. Ponceau S staining was used as the loading control.

In the present study we investigated the effect of Erg11p external inhibition by treating *C. albicans* WT strain with fluconazole on sterol profile, *ERG11* gene expression, plasma membrane (PM) fluidity and potential, CDR1 gene expression and Cdr1p efflux activity (Figures S4). Similarly to *ERG11* gene deletion, the fluconazole treatment had rigidifying and depolarizing effect on the PM (Figures S4C and S4D). However, it was concluded that those effects occurred despite dissimilar effect on sterol profile (Figure S4A and S4B). Additionally, similarly to *ERG11* gene deletion, the fluconazole treatment induced *CDR1* gene expression but reduced its activity, as measured by external rhodamine 6G (R6G) fluorescence intensity (Figure S4E and S4F).

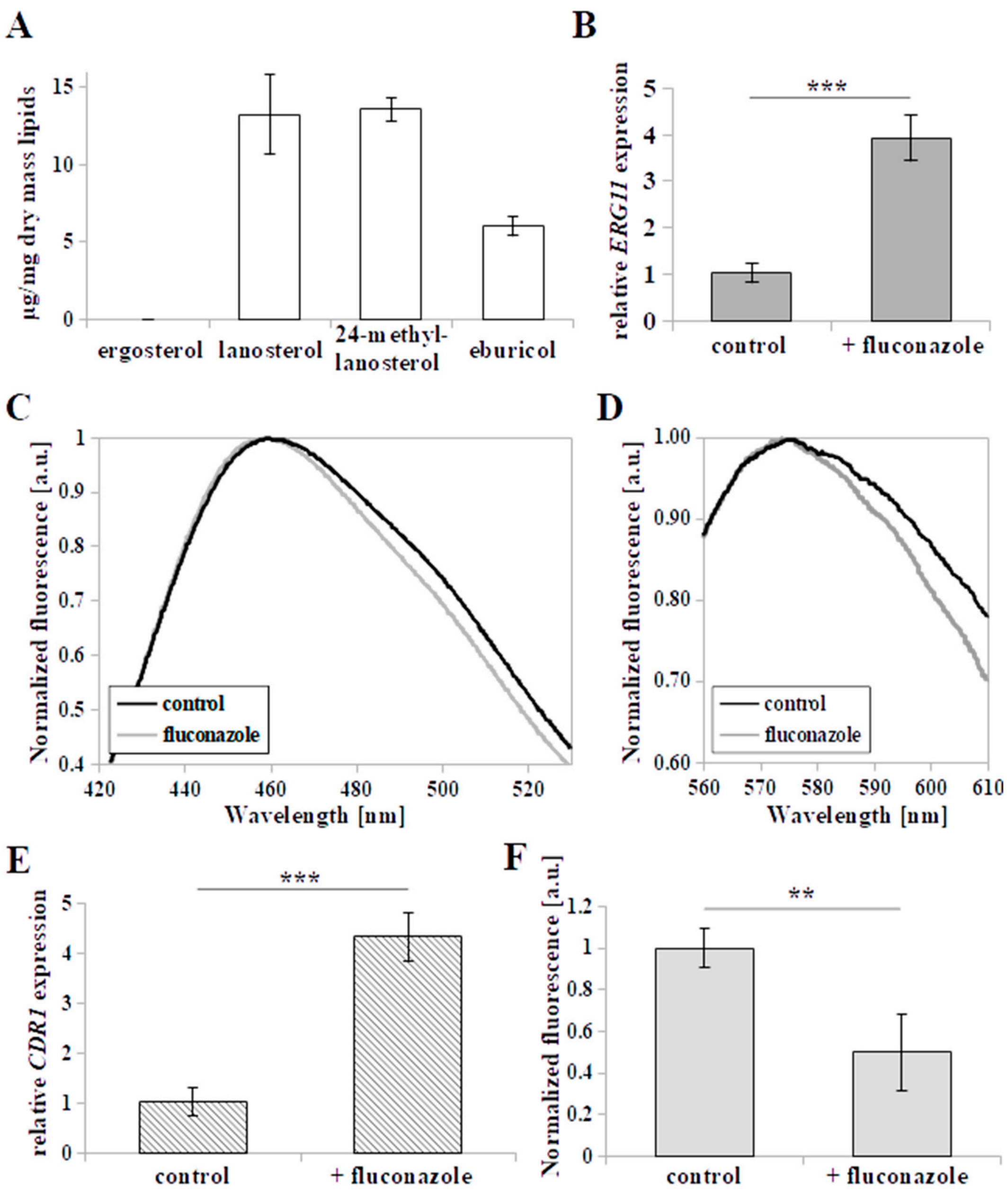

Figure S4. (**A**) Sterol metabolites (μg/mg dry mass of isolated lipids, means ± SD, *n* =3) in *C. albicans* CAF2-1 (WT) grown for 24 h in presence of 4 μg/mL fluconazole. (**B**) Relative *ERG11* gene expression in *C. albicans* CAF2-1 strain grown for 24 h in presence of 4 μg/mL fluconazole; gene expression levels are reported as means of $2^{-\Delta\Delta CT}$ values (*n* = 6) ± SD; normalised to 1 for control conditions (non-treated with fluconazole). (**C**) Fluorescent spectra of laurdan incorporated into plasma membrane (PM) of *C. albicans* CAF2-1 strain grown for 8 h in presence of 8 μg/mL fluconazole, each spectrum is averaged (*n* = 6). (**D**) Fluorescent spectra of di-4-ANEPPS incorporated into PM of *C. albicans* CAF2-1 strain grown for 8 h in presence of 8 μg/mL fluconazole, each spectrum is averaged (*n* = 6). (**E**) Relative *CDR1* gene expression in *C. albicans* CAF2-1 grown for 24 h in presence of 4 μg/mL fluconazole; gene expression levels as means of $2^{-\Delta\Delta CT}$ values (*n* = 6) ± SD; normalised to 1 for control conditions. (**F**) Cdr1p-dependent rhodamine 6G (R6G) efflux in *C. albicans* CAF2-1 grown for 24 h in presence of 4 μg/mL fluconazole shown as normalised (=1 for control conditions) fluorescence intensity of extracellular R6G (means ± SD, *n* = 3). Statistical analysis was performed by in comparison to corresponding control experiments (*, $p < 0.05$; **, $p < 0.01$; ***, $p < 0.001$).

The effect of external inhibition of Erg11p by fluconazole treatment on Cdr1p-GFP localization in CAF2-1 strain was evaluated in 8 hr of growth (Figure S5). We concluded that, similarly to *ERG11* gene deletion, the fluconazole treatment causes Cdr1p-GFP mislocalization to vacuoles.

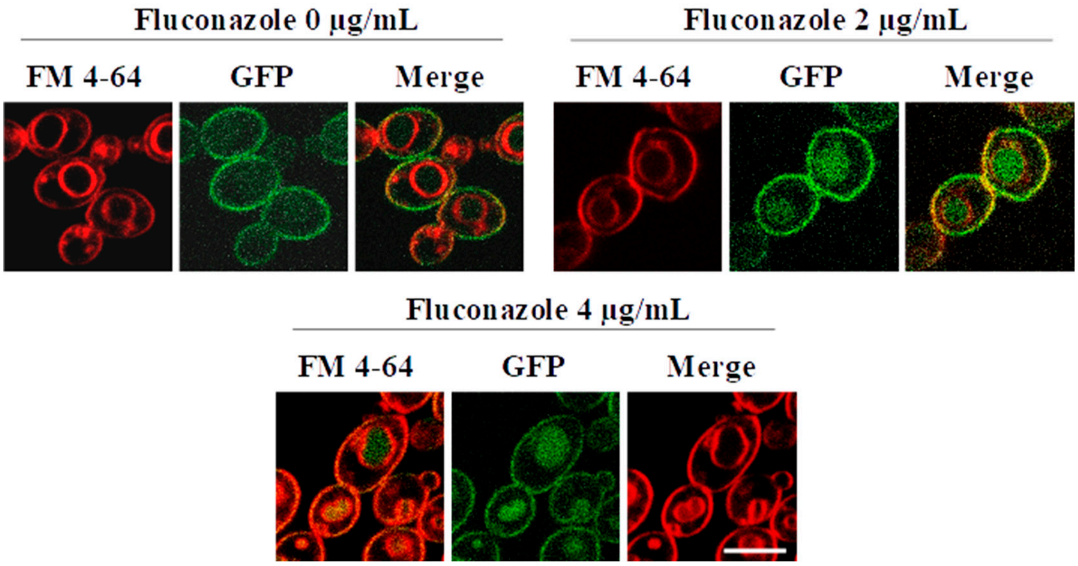

Figure S5. Confocal micrographs of vacuolar membrane staining (FM 4-64) and subcellular localization of Cdr1-GFP protein in *C. albicans* strains ASCa1 (*CDR1-GFP*) grown until 8 h in presence of 0, 2 or 4 μg/mL fluconazole. A merged image of the GFP-tagged protein and FM 4-64 staining is shown in the third column. Scale bar = 5 μm.

© 2019 by the authors. Submitted for possible open access publication under the terms and conditions of the Creative Commons Attribution (CC BY) license (http://creativecommons.org/licenses/by/4.0/).
